# Phase One of a Global Evaluation of Suction-Based Airway Clearance Devices in Foreign Body Airway Obstructions: A Retrospective Descriptive Analysis

**DOI:** 10.3390/ijerph19073846

**Published:** 2022-03-24

**Authors:** Cody L. Dunne, Selena Osman, Kayla Viguers, Ana Catarina Queiroga, David Szpilman, Amy E. Peden

**Affiliations:** 1 Department of Emergency Medicine, University of Calgary, Calgary, AB T2N2T9, Canada; 2 International Drowning Researchers’ Alliance, Kuna, ID 83634, USA; queirogaac@gmail.com (A.C.Q.); david@szpilman.com (D.S.); a.peden@unsw.edu.au (A.E.P.); 3 Cumming School of Medicine, University of Calgary, Calgary, AB T2N4N1, Canada; sosma@ucalgary.ca; 4 Faculty of Medicine, Memorial University of Newfoundland, St. John’s, NL A1C5S7, Canada; kbv476@mun.ca; 5 EPIUnit, Instituto de Saúde Pública da Universidade do Porto, 4050-600 Porto, Portugal; 6 Laboratory for Integrative and Translational Research in Population Health (ITR), 4200-319 Porto, Portugal; 7 Brazilian Lifesaving Society (SOBRASA), Barra da Tijuca, Rio de Janeiro 22631-004, Brazil; 8 School of Population Health, Faculty of Medicine, University of New South Wales, Sydney, NSW 2052, Australia; 9 College of Public Health, Medical and Veterinary Sciences, James Cook University, Townsville, QLD 4811, Australia

**Keywords:** foreign body airway obstruction, anti-choking, prehospital, basic life support, resuscitation

## Abstract

Background: Choking is a prevalent source of injury and mortality worldwide. Traditional choking interventions, including abdominal thrusts and back blows, have remained the standard of care for decades despite limited published data. Suction-based airway clearance devices (ACDs) are becoming increasingly popular and there is an urgent need to evaluate their role in choking intervention. The aim of this study was to describe the effectiveness (i.e., resolution of choking symptoms) and safety (i.e., adverse events) of identified airway clearance devices interventions to date. Methods: This retrospective descriptive analysis included any individual who self-identified to manufacturers as having used an ACD as a choking intervention prior to 1 July 2021. Records were included if they contained three clinical variables (patient’s age, type of foreign body, and resolution of choking symptoms). Researchers performed data extraction using a standardized form which included patient, situational, and outcome variables. Results: The analysis included 124 non-invasive (LifeVac©) and 61 minimally invasive (Dechoker©) ACD interventions. Median patient age was 40 (LifeVac©, 2–80) and 73 (Dechoker©, 5–84) with extremes of age being most common [<5 years: LifeVac© 37.1%, Dechoker© 23.0%; 80+ years: 27.4%, 37.7%]. Food was the most frequent foreign body (LifeVac© 84.7%, Dechoker© 91.8%). Abdominal thrusts (LifeVac© 37.9%, Dechoker© 31.1%) and back blows (LifeVac© 39.5%, Dechoker© 41.0%) were often co-interventions. Resolution of choking symptoms occurred following use of the ACD in 123 (LifeVac©) and 60 (Dechoker©) cases. Three adverse events (1.6%) were reported: disconnection of bellows/mask during intervention (LifeVac©), a lip laceration (Dechoker©), and an avulsed tooth (Dechoker©). Conclusion: Initial available data has shown ACDs to be promising in the treatment of choking. However, limitations in data collection methods and quality exist. The second phase of this evaluation will be an industry independent, prospective assessment in order to improve data quality, and inform future choking intervention algorithms.

## 1. Introduction

Despite being preventable, foreign body airway obstructions (FBAO, choking) are a significant source of injury and mortality worldwide [1,2,3,4,5]. In the United States alone, over 5000 deaths from choking are reported annually [6]. Further, for each pediatric fatality due to choking, it is reported that 110 non-fatal events present to emergency departments, of which 10% result in-hospital admission [7]. Extrapolating to the entire lifespan, choking injuries result in a considerable burden on global healthcare systems and more importantly, preventable injury and loss of life.

Prehospital choking interventions have remained largely unchanged for several decades and consist of a combination of abdominal thrusts, back blows and chest compressions or thrusts [8,9,10]. However, the evidence for these techniques is almost entirely case series data and there is uncertainty over which intervention (if any) is superior [8].

Externally applied suction-based airway clearance devices (ACDs) have been introduced as a possible alternative when traditional techniques are unsuccessful [11,12]. Two types are currently marketed, those which are non-invasive (e.g., LifeVac©, LifeVac LLC, Nesconset, New York, NY, USA) and those which are minimally invasive (e.g., DeChoker©, LLC, Wheat Ridge, CO, USA) [11,12]. A third device is in the pre-market, fundraising phase [13]. Despite their increasing popularity, there is not yet sufficient data available in academic literature to fully assess their safety and effectiveness [8,9,14].

There is an urgent need for more data in this field as choking remains a significant cause of death and injury [1,2,3,4,5]. A new intervention for prehospital lay rescuers and emergency medical service (EMS) teams would be welcomed, provided it can be demonstrated to not cause harm and assist with choking relief. As the public gains awareness and the availability of ACDs increases, resuscitation councils who determine choking treatment guidelines must be able to clearly comment on their role [11,12]. 

This retrospective analysis is the first phase in a multi-method global evaluation of ACDs, which aims to fill this knowledge gap [15]. The objective of this study is to describe what situational and patient factors have been identified in cases where ACDs were used, as well as report on patient outcomes. These results will inform the next phase of this evaluation which will be the development of a prospective, industry independent database of ACD cases. 

## 2. Methods

This is a retrospective study evaluating ACD interventions from 1 January 2016, to 30 June 2021, globally. The start date represents the earliest report of an ACD intervention to device manufacturers. A detailed description of the study development and methodology has been published previously [15]. A brief summary is presented below. The study was approved by the Human Research Ethics Committee (HREC) of the University of New South Wales (HC210242) on 25 May 2021.

## 3. Data Collection

Participants in the study include individuals who self-identified to device manufacturers as having used an ACD on someone choking between 1 January 2016, and 1 July 2021. A waiver of consent for the secondary use of a dataset was granted by the HREC. Device manufacturers have developed their own methods to allow customers who have used their ACD on a choking individual to report their experience and they agreed to provide all cases reported to them, regardless of outcome, for this initial evaluation. Due to the novelty of ACDs and relative rarity of interventions, investigation into a single health system was not feasible for this preliminary work and this represents the population of all cases reported to date.

**Figure 1:**
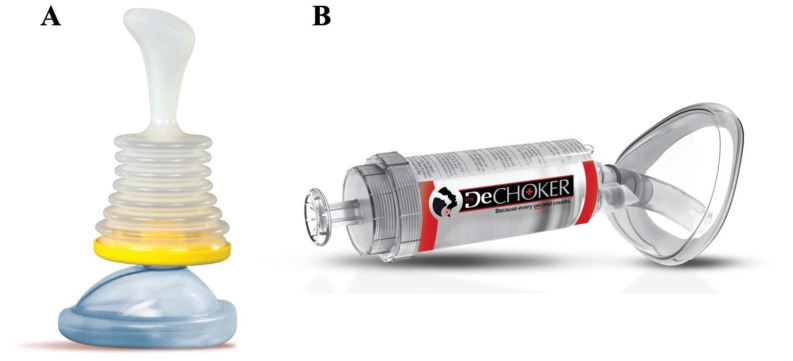
(**A**) LifeVac© airway clearance device (**B**) DeChoker© airway clearance device [images supplied by the respective manufacturers with permission to include].

Presently, two manufacturers are primarily responsible for the production of suction-based ACDs around the world. Each represents a different ACD type, and although they have a similar goal, the contrasting designs make it important to distinguish datasets. Non-invasive ACDs have no intraoral component, whereas minimally invasive do. These both differ from invasive (or deep) suction devices (e.g., Laerdal© V-Vac^®^) which have no external facemask that anchors the device and therefore can extend deep into the airway [16]. Figure 1 displays both types of ACD devices.

### 3.1. Non-Invasive ACD

LifeVac LLC produces the LifeVac© ACD [11]. It consists of a facemask attached to compressible bellows and a one-way valve. The LifeVac database of ACD interventions relies primarily on their online reporting system (Supplementary File S1, Table S1) [17]. All purchasers are informed of this system in the shipping package, and it is promoted on their social media platforms. Once a user reports their experience, an administrator from one of their regional offices is notified and subsequently follows up with each user to confirm the details of the choking event and validate the report submission.

A standardized reporting form is used to record data from each clinical intervention (Supplementary File S1, Table S2). No intervention is recorded into the database until an administrator connects with the user. LifeVac LLC provided all their collected data (regardless of outcome) to the research team electronically from their compiled clinical evaluation reports. 

### 3.2. Minimally Invasive ACD

DeChoker LLC produces the DeChoker© ACD [12]. It is designed with a face mask attached to a cylinder with a plunger. In the face mask is a 3-inch (7.6 cm) tube that is directed into the oropharynx to act as a tongue depressor. The tube also is the passageway for the negative pressure suction and has a diameter of 0.75-inch (1.9 cm). 

The data obtained and how they are collected differs depending on geographic region. Outside of the United States of America (USA), most sales are directed towards care facilities via local distributors. Care facilities are encouraged to report any interventions regardless of outcome back to the distributors who then inform DeChoker LLC. In the USA, while some cases are also from care facilities, others are from individuals who self-identify directly to DeChoker either via an online reporting system or the device’s social media platforms. 

Regardless of region, once identified, a member of the DeChoker team attempts to follow up with users to confirm details and validate the database entry. No standardized reporting form is used consistently to record data by administrators. Dechoker LLC provided their data to the research team in several electronic documents consisting of intervention reports from different global regions (namely North America and Europe) and social media posts.

### 3.3. Variables

Key demographical, clinical and safety data were categorized for analysis. Age was classified in six groups for analysis: under 1, 1 to 5, 6 to 18, 19 to 64, 65 to 80, and over age 80. Pre-existing medical conditions were classified into five groups: cardiovascular disease, respiratory disease, physical disability, neurocognitive disorder, and other. 

Choking severity was classified into three categories: (a) partial (also known as incomplete or mild) is defined as when the patient can cough forcefully, cry, speak or still perform good air exchange; (b) complete (also known as severe) is defined as when the patient has a weak ineffective cough, unable to speak or cannot perform good air exchange (e.g., making only high pitch noise); and (c) unresponsive [18,19].

Choking location was grouped as: home, school/daycare, nursing home, or other. Type of foreign body was classified as: food, toy, or other. Non-ACD interventions were separated into abdominal thrusts (previously known as Heimlich maneuver), back blows, chest thrusts or compressions, finger sweep or none. ACD user profile categories were relative, healthcare worker, self, or other. An attempt with the ACD was defined as one plunge-release cycle.

All variables had a planned ‘not recorded’ option included as data completeness was anticipated to be variable due to the differences in intervention follow up and record keeping amongst manufacturers.

### 3.4. Outcomes

In the current study, both effectiveness and safety were described. Effectiveness was determined as cases where no further choking intervention was required (i.e., resolution of symptoms, yes/no) after use of the ACD, and survival (alive/dead) [20]. No further choking intervention being deemed needed by the rescuer was used as a surrogate marker of effectiveness as relief of obstruction could not be directly assessed. Safety was assessed by summarizing adverse events. Adverse events could be patient-related (e.g., injury to face from device use) or device-related (e.g., ACD broke when being applied). 

### 3.5. Data Analysis

Two researchers (SO, KV) reviewed the raw clinical data and performed data extraction via a standardized form (Supplementary File S2). Subsequently, another researcher (CD) reviewed the extracted data and performed a secondary check of a random 20% of the entries for accuracy and consistency amongst the two extractors.

It was decided *a priori* that, for a record to be included in the final analysis, three clinical data points were required: the patient’s age, a description of the foreign body material and commentary on the primary outcome. There were 140 LifeVac© interventions recorded, of which 124 (88.6%) were eligible for inclusion. There were 111 Dechoker© interventions recorded, of which 61 (55.0%) were eligible for inclusion. The one exception to this was for adverse events. For complete transparency, we decided to review all the cases included in the database (even those not meeting inclusion criteria) so that all potential adverse events were known.

Descriptive statistics were performed to summarize the data. Age and number of ACD attempts were reported as median and interquartile range (IQR). Categorical data were expressed as frequency distributions (*n* (%)).

## 4. Results

There have been 124 LifeVac© and 61 Dechoker© interventions (which met inclusion criteria for analysis) since 2016. Table 1 summarizes the characteristics of the person experiencing the FBAO. 

LifeVac© ACDs have a wide representation across the age span (median age, IQR = 40, range = 2–80 years) with about one-third of the interventions being younger than five years and another third aged 65 years and older. Pre-existing medical co-morbidities were common (59.6% having at least one), with neurocognitive disorders (38.7%) and physical disabilities (25.8%) being the most prevalent (Table 1). They were deployed for both partial (27.4%) and complete (41.9%) FBAO. For these ACDs, choking events were much more common at home (22.6%) or long-term care facilities (36.3%) compared to schools/daycares (0.8%).

Dechoker© ACDs were commonly used in a more elderly population (median age, IQR = 73, range = 5–84 years) with over half being 65 years and older. Medical comorbidities were documented infrequently (18.0%), though neurocognitive conditions were also the most prevalent (11.5%). Home (34.4%) and long-term care (39.3%) were the most common geographic locations, compared to schools (0.0%).

For both ACD types, females were more commonly treated (LifeVac©-53.2%; Dechoker©-59.0%) and a relatively small number of patients had a known history of dysphagia or aspiration (13.7%; and 4.8%). Similarly, food was the predominant foreign body for both ACD types (84.7%; and 91.8%). Besides food and toys, other foreign bodies included: plastic, medication pills, saliva/mucus/phlegm, emesis, fluid, and coins. Table 2 further summarizes the FBAO details. 

The pattern of non-ACD interventions were similar in both groups. Abdominal thrusts (LifeVac©-37.9% and Dechoker©-31.1%) and back blows (39.5% and 41.0%) were frequently utilized, while chest thrusts or compressions (3.2% and 3.3%) and finger sweeps (7.3% and 6.6%) were rarer. The median number of ACD attempts required before choking was considered resolved by the rescuer was two for both types. Table 3 presents data regarding the choking interventions and outcomes. 

LifeVac© ACDs were the last intervention in 123 cases (of 124) and all patients subsequently survived. EMS was called in 42.7% of cases, and subsequent hospital admission occurred in 13.6%. There was one adverse outcome where an untrained individual attempted to use the device, but the bellows/mask disconnected prior to use due to incorrect assembly. The patient had a traditional technique subsequently applied and survived the event.

Dechoker© ACDs were the last intervention in 60 cases (of 61). All patients survived, except in one case where FBAO was relieved, but survival was not confirmed. EMS was called in 35.1% of cases, and subsequent hospitalization occurred in 2.8%. Two adverse events were reported. One where the user had difficulty inserting the tongue depressor into the panicked patient’s mouth when they were conscious, and as a result, the patient had a cut on their lip from the device. The second was where a person’s tooth was avulsed when the tongue depressor was inserted into the oropharynx.

## 5. Discussion

Airway clearance devices appear to have the potential to help save lives. This study is the first of a multi-phase global evaluation of ACDs that aims to determine their effectiveness and clarify their role (if any) in future choking intervention algorithms [15]. Prior to this study, most published data were limited to mannequin studies, case reports with few entries, or only focused on a subset of the population [8,9,14,21,22]. This study included all ACD intervention data available, incorporating all ages from all regions of the world. 

The initial data described are promising. LifeVac© and Dechoker© ACDs were the last intervention before resolution of choking symptoms in 123 and 60 cases, respectively. However, current data collection and quality processes require further research before definite conclusions are made. 

Data collection via self-reporting is required presently as ACDs are not prevalent enough to investigate a particular health region for interventions. Self-reporting is known to predispose the results to exceptional (successful) cases [23,24,25]. This makes it inappropriate to conclude that the effectiveness of these devices is 99.2% (LifeVac©) and 98.4% (Dechoker©) as we have no way to determine the true denominator (i.e., total number of times an ACD has been utilized in a FBAO). Further, self-reporting to manufacturers is much less likely to occur in cases where ACDs were used and did not work [23,24,25].

Data quality also limits interpretation of this data. The self-reported data are not supported by medical records and were not collected by trained medical professionals. This results in important details being omitted from the data. For example, 35 patients were reported as unresponsive during ACD use, but only 10 had EMS activated. Medical oversight would improve recognition of conflicting information, resulting in further questioning and clarity in our understanding of the situation. 

Like all choking intervention research, confirmation of the severity of the obstruction is challenging because it relies on bystander interpretation of the patient’s condition and symptoms. This data point is important however because traditional teaching recommends only encouraged forceful coughing for partial cases, due to the potential for harms or worsening the obstruction from interventions [18,19]. In our study, both LifeVac© (38.7%) and Dechoker© (68.9%) ACDs had a significant proportion of cases which were classified as a partial obstruction or unknown severity. It is possible that the cases with a partial obstruction may not have required any intervention to clear. In these situations, it is unclear if the ACDs truly prevented further deterioration or just appeared to have benefit due to early use in mild cases.

Despite the early application of ACDs in some cases, we fortunately found that reported adverse outcome rates were low and relatively benign for ACDs compared to those following other choking interventions such as abdominal thrusts or chest compressions (e.g., organ rupture and vascular injury) [8]. A recent cadaver evaluation, conducted without industry involvement, found injury to the tongue following use of the Dechoker© [26]. This was identified in our human study as well. No injury was found due to LifeVac in the cadaver evaluation [26]. Other studies have limited information on safety [8,9,14,21,22]. Unfortunately, self-reporting has been shown to have poor sensitivity for detecting adverse events [24,25], which is compounded in this study by limited patient follow up and the data quality concerns described previously. Any future evaluation of these devices requires specific questioning around potential adverse events from medical personnel to improve sensitivity.

**Figure 2:**
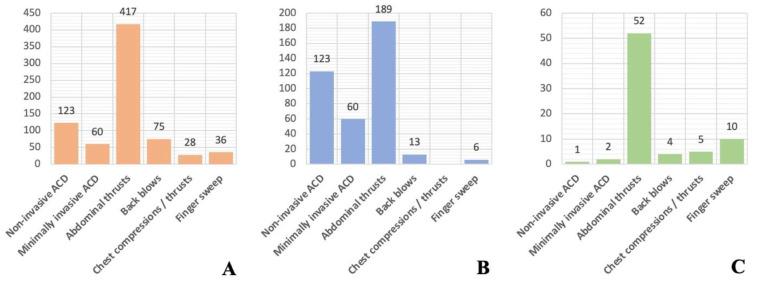
Reported counts in academic literature of effectiveness and safety outcomes for airway clearance devices and traditional FBAO interventions: (**A**) Relief of FBAO (**B**) Survival* (**C**) Adverse events [8,9]. * Chest compressions/thrusts had survival with good neurological outcome reported, not survival.

The criticism of these data, however, needs to be interpreted in the context of what is available for other choking interventions. Current treatment recommendations for traditional interventions are based on only one cross-sectional study, and six case series published between 1979 and 2017 [8,9]. Figure 2 compares the number of published cases reporting relief of FBAO and adverse events for ACDs for traditional interventions. The two studies that contribute the largest amount of data also use a self-reporting methodology [27,28]. It is clear we need more investigation and better data for all choking interventions, not just ACDs. 

The cases in the current study should not change current practice. However, they should encourage researchers and medical professionals to ask more questions and investigate further. LifeVac© and Dechoker© ACDs were used in 123 and 59 situations, respectively, where a bystander believed someone was choking and were the last intervention before the choking symptoms resolved. In 109 and 50 of these cases, other traditional interventions had been attempted prior but were not deemed by the rescuer to relieve the symptoms of choking. The potential of a novel layperson treatment for choking deserves attention, especially in the absence of high-quality data for other techniques. 

To improve our present understanding, attention must be paid to data collection and quality. While a self-reporting methodology is inevitable presently, data that are prospectively collected, industry-distanced, with medical oversight and follow up, will shed more light on the role ACDs could play in the treatment of choking. One such study is ongoing, though multiple investigations are needed [15].

## 6. Conclusions

Non-invasive and minimally invasive ACDs are novel interventions with positive initial findings. Prospective evaluation, independent of manufacturers, that improves data quality will further determine the devices respective roles in the response of healthcare workers and layrescuers to a choking person.

## Figures and Tables

**Table 1:** Characteristics of patients with a foreign body airway obstruction intervened by an airway clearance device.

		Non-Invasive ACD (LifeVac©) N = 124	Minimally Invasive ACD (DeChoker©) N = 61
Patient Gender (*n*, %)			
	M	56 (45.2)	24 (39.3)
	F	66 (53.2)	36 (59.0)
	Not recorded	2 (1.6)	1 (1.6)
Patient age (median, IQR)		40 (2–80)	73 (5–84)
Patient age groups (*n*, %)			
	0–1 years	19 (15.3)	5 (8.2)
	1–5 years	27 (21.8)	9 (14.8)
	6–18 years	9 (7.3)	8 (13.1)
	18–64 years	22 (17.7)	6 (9.8)
	65–80 years	13 (10.9)	10 (16.4)
	80+ years	34 (27.4)	23 (37.7)
Pre-existing medical conditions (*n*, %)			
	Cardiovascular disease	4 (3.2)	0 (0.0)
	Neurocognitive disorder	48 (38.7)	7 (11.5)
	Physical disability	32 (25.8)	2 (3.2)
	Respiratory disease	1 (0.8)	1 (1.6)
	Wheelchair use	18 (14.5)	2 (3.2)
	Other	16 (12.9)	1 (1.6)
	None	47 (37.9)	- *
	Not recorded	8 (6.5)	48 (78.7)
Known history of dysphagia or aspiration (*n*, %)			
	Yes	17 (13.7)	3 (4.8)
	Not recorded	107 (84.3)	58 (95.2)

ACD = airway clearance device. * Not able to be calculated as these data were not routinely collected and only identified if volunteered by report provided.

**Table 2:** Characteristics of the foreign body airway obstruction in patients intervened with an airway clearance device.

		Non-Invasive ACD LifeVac© (N = 124)	Minimally Invasive ACD Dechoker© (N = 61)
Severity of FBAO (*n*, %)			
	Partial	34 (27.4)	5 (8.2)
	Complete	52 (41.9)	8 (13.1)
	Unresponsive	24 (19.4)	11 (18.0)
	Not recorded	14 (11.3)	37 (60.7)
Geographical location of FBAO (*n*, %)			
	Home	28 (22.6)	21 (34.4)
	School/Daycare	1 (0.8)	0 (0.0)
	Long-term care facility/Nursing home	45 (36.3)	24 (39.3)
	Other	11 (8.9)	2 (3.3)
	Not recorded	39 (31.5)	14 (23.0)
Foreign body (*n*, %)			
	Food	105 (84.7)	56 (91.8)
	Toy	1 (0.8)	1 (1.6)
	Other	18 (14.5)	4 (6.6)

ACD = airway clearance device; FBAO = foreign body airway obstruction.

**Table 3:** Intervention and outcome data for patients with a FBAO intervened by an airway clearance device.

		Non-Invasive ACD LifeVac© (N = 124)	Minimally Invasive ACD Dechoker© (N = 61)
Pre-ACD Intervention			
	Abdominal thrusts	47 (37.9)	19 (31.1)
	Back blows	49 (39.5)	25 (41.0)
	Chest thrusts or compressions	4 (3.2)	2 (3.3)
	Finger / mouth sweep	9 (7.3)	4 (6.6)
	Multiple interventions	25 (20.2)	15 (24.6)
	No intervention	11 (8.9)	10 (16.4)
	Not recorded	31 (25.0)	17 (27.9)
ACD User			
	Relative	42 (33.8)	22 (36.1)
	Healthcare worker	12 (9.7)	2 (3.3)
	Self	1 (0.8)	0 (0.0)
	Other	10 (8.1)	21 (34.4)
	Not recorded	59 (47.6)	16 (26.2)
Median number of ACD attempts to FBAO relief (IQR; range)		2 (1–3; 1–12)	2 (1–4; 1–12)
Effectiveness Outcomes			
	No Further Intervention Required Post-ACD	123	60
	Survival	123	59 *
Safety Outcomes			
	EMS called	33 (42.9) ^1^	13 (35.1) ^2^
	Hospital admission	9 (13.6) ^3^	1 (2.8) ^4^
	Adverse events reported	1 (1.1) ^5^	2 (5.4) ^2^

ACD = airway clearance device; FBAO = foreign body airway obstruction. Missing values: ^1^ *n* = 77; ^2^ *n* = 37; ^3^ *n* = 66; ^4^ *n* = 36; ^5^ *n* = 94. * One record did not confirm the survival status.

## Data Availability

Restrictions apply to the availability of these data. Data were obtained from manufacturers and are available with the permission of the respective organizations.

## Supplementary Materials

The following supporting information can be downloaded at: https://www.mdpi.com/article/10.3390/ijerph19073846/s1, Table S1: LifeVac© online use reporting form data fields (16); Table S2: LifeVac© clinical evaluation report data fields; Supplementary File S2—Standardized reporting tool used by researchers for data extraction.

## References

[1] Health Canada (2011). Canadian Injury Data: Mortality 2005 and Hospitalizations, 2001–2005.

[2] Injury Facts (2017). Preventable Death and Death Rates per 100,000 Population in the Home and Community by Cause and Age Group, United States.

[3] Institute for Health Metrics and Evaluation (2019). Disease and Risk Factor Summaries: Pulmonary Aspiration and Foreign Body in Airway.

[4] Fridman L., Fraser-Thomas J., Pike I., Macpherson A.K. (2018). An interprovincial comparison of unintentional childhood injury rates in Canada for the period 2006–2012. Can. J. Public Health.

[5] Norii T., Igarashi Y., Sung-Ho K., Nagata S., Tagami T., Yoshino Y., Hamaguchi T., Maejima R., Nakao S., Albright D. (2020). Protocol for a nationwide prospective, observational cohort study of foreign-body airway obstruction in Japan: The MOCHI registry. BMJ Open.

[6] Injury Facts (2019). Preventable-Injury-Related Deaths by Sex, Age and Cause, United States, 1999–2019.

[7] Centers for Disease Control and Prevention (2002). Non-fatal choking-related episodes among children—United States, 2001. MMWR.

[8] Couper K., Abu Hassan A., Ohri V., Patterson E., Tang H.T., Bingham R., Olasveengen T., Perkins G.D. (2020). Removal of foreign body airway obstruction: A systematic review of interventions. Resuscitation.

[9] Couper K., Abu Hassan A., Ohri V., Patterson E., Tang H.T., Bingham R., Perkins G.D., Avis S., Brooks S., Castren M. (2020). Consensus on Science with Treatment Recommendations: Removal of Foreign Body Airway Obstruction.

[10] Soroudi A., Shipp H.E., Stepanski B.M. (2007). Adult foreign body airway obstruction in the prehospital setting. Prehosp. Emerg. Care.

[11] LifeVac LLC (2021). Lifevac.

[12] DeChoker LLC (2021). Dechoker.

[13] ExtraLife (2021). Lifewand.

[14] Dunne C., Peden A., Queiroga A., Gonzalez C.G., Valesco B., Szpilman D. (2020). A systematic review on the effectiveness of anti-choking suction devices and identification of research gaps. Resuscitation.

[15] Dunne C.L., Queiroga A.C., Szpilman D., Viguers K., Osman S., Peden A.E. (2022). A protocol for the prospective evaluation of novel suction-based airway clearance devices in the treatment of foreign body airway obstructions. Cureus.

[16] Laerdal Medical (2021). V-Vac™ Manual Suction Unit.

[17] LifeVac LLC (2021). LifeVac Saved a Life Report.

[18] American Heart Association (2000). Part 3: Adult basic life support. Circulation.

[19] Perkins G.D., Handley A.J., Koster R.W., Castrén M., Smyth M.A., Olasveengen T., Monsieurs K.G. (2015). European Resuscitation Council Guidelines for Resuscitation 2015. Section 2. Adult basic life support and automated external defibrillation. Resuscitation.

[20] Safar P., Grenvik A., Safar P. (1981). Resuscitation and brain ischemia. Brain Failure and Resuscitation.

[21] Bhanderi B.G., Hill S.P. (2020). Evaluation of DeChoker, an Airway Clearance Device (ACD) Used in Adult Choking Emergencies within the Adult Care Home Sector: A Mixed Methods Case Study. Front. Public Health.

[22] Gal L.L., Pugleisi P., Peterman D. (2020). Resuscitation of choking victims in a pediatric population using a novel portable non-powered suction device: Real world data. Pediatr. Ther..

[23] Smith M. (2012). Biased Sample and Extrapolation, Common Mistakes in Using Statistics: Spotting and Avoiding Them. https://web.ma.utexas.edu/users/mks/statmistakes/biasedsampling.html.

[24] Hoopeer A.J., Tiballs J. (2014). Comparison of a Trigger Tool and voluntary reporting to identify adverse events in a paediatric intensive care unit. Aneasth. Intensive Care.

[25] Weingart S.N., Callanan L.D., Ship A.N., Aronson M.D. (2001). A physician-based voluntary reporting system for adverse events and medical errors. J. Gen. Intern. Med..

[26] Ramaswamy A.T., Done A., Solis R., Evangelista L., Belafsky P. The efficacy of two commercially available devices to relieve acute foreign body aspiration. Proceedings of the Annual Meeting ABEA.

[27] Redding J.S. (1979). The choking controversy: A critique of the Heimlich. Crit. Care Med..

[28] Heimlich H.J. (1975). A Life-Saving Maneuver to Prevent Food Choking. JAMA.

