# Supplementary material for: Phase One of a Global Evaluation of Suction-Based Airway Clearance Devices in Foreign Body Airway Obstructions: A Retrospective Descriptive Analysis
Source: Int J Environ Res Public Health. 2022 Mar 24;19(7):3846. doi: 10.3390/ijerph19073846 (PMC8998090; doi:10.3390/ijerph19073846)
Supplement: Supplementary file 1 [file ijerph-19-03846-s001.zip › ijerph-1633365-supplementary.pdf]

# Supplementary File S1: Data collection tools used by device manufacturers

**Table S1.** LifeVac © online use reporting form data fields (16).

| **Text Field** | **Description Provided (if any)** | **Type of response** |
|---|---|---|
| Date of Incident | | Date entry |
| Location of Incident | | Free text |
| Patient | Name of patient | Free text |
| Any known medical conditions | | Free text |
| Object that created the blockage | | Free text |
| Partial or total blockage? (if known) | | Free text |
| Was the Heimlich maneuver / back blows performed? | | Yes or No choice |
| Was patient conscious at time of device use? | | Yes or No choice |
| Number of times LifeVac was used? (Place, Push, Pulled) | | Free text |
| Outcome | | Free text |
| Do you feel LifeVac saved the life of the person choking? | | Free text |
| Your contact information to receive free LifeVac* | Name, phone, email address | Free text |
| Your Address | The user's address | Free text |
| How did you hear about LifeVac? | | Free text |

**Table S2.** LifeVac © clinical evaluation report data fields.

| |
|---|
| Date |
| Location |
| Patient age / sex |
| Medical conditions |
| Nature / type of blockage (if known) |
| Partial or total blockage (if known) |
| BLS protocol first followed? |
| Patient conscious at time of device use? |
| Position of patient when LifeVac applied? |
| Number of uses of LifeVac attempted? |
| Hazard successfully dislodged with LifeVac? |
| Duration of patient choking until recovery? |
| Outcome |
| Did patient receive medical examiniation afterwards due to BLS being performed? |
| What complications were found, if any, from examination? |

## Supplementary File S2 – Standardized reporting tool used by researchers for data extraction

| Data Field or Question | Response Type | Answer choices (if applicable) |
|---|---|---|
| Assigned Number (by LifeVac LLC or DeChoker LLC) | Numerical | |
| Age | Numerical | |
| Date of Incident | Free – text | |
| Gender | Multiple choice | Male, female, other, not recorded |
| Geographical location of FBAO | Multiple choice | Home, school/daycare, long term care/nursing home, other, not recorded |
| Medical comorbidities | Free – text | |
| Types of medical comorbidities | Multiple choice | Cardiac, respiratory, physical disability, neurocognitive disorder, other |
| History of dysphagia / chewing / aspiration difficulty | Multiple choice | Yes, no, not recorded |
| Wheelchair use | Multiple choice | Yes, no, not recorded |
| Foreign body description | Free – text | |
| Foreign body type | Multiple choice | Food, toy, other |
| Degree of obstruction | Multiple choice | Mild (partial, incomplete), severe (complete), not recorded |
| Data provided in report that supports degree of obstruction | Free - text | |
| Level of consciousness of patient | Multiple choice | Alert, altered, unresponsive, not recorded |
| Duration (approximate) of FBAO | Numeric | |
| Were traditional techniques used before or after ACD use? | Multiple choice | Yes, no, not recorded |
| Description of non-ACD interventions (what technique, how many, before or after) | Free – text | |
| Traditional techniques used pre-ACD (Select all that apply) | Multiple choice | Abdominal thrusts, back blows, chest compressions / thrusts, finger sweep, other, none |

| | | |
|---|---|---|
| CPR performed before or after ACD | Multiple choice | Yes, no, not recorded |
| Description of ACD user | Free – text | |
| Training of ACD user | Multiple choice | Healthcare worker, first aid trained, no first aid training, not recorded |
| Number of ACD attempts (one push/pull cycle = one attempt) | Numerical | |
| Patient position when ACD used | Free – text | |
| Did ACD relieve the FBAO? | Multiple choice | Yes, no, not recorded |
| If the ACD did not relieve the FBAO, what did? | Free – text | |
| Did the patient survive? | Multiple choice | Yes, no, not recorded |
| Was EMS called? | Multiple choice | Yes, no, not recorded |
| Did the patient go to the hospital? | Multiple choice | Yes, no, not recorded |
| Was the patient admitted to hospital? | Multiple choice | Yes, no, not recorded |
| Were there any adverse events? | Multiple choice | Yes, no, not recorded |
| Describe the adverse events. | Free – text | |
| Summarize the details of the incident and outcome | Free – text | |
